# Functional connectivity density of different suicidal behaviors in adolescents with major depressive disorders

**DOI:** 10.3389/fpsyt.2024.1491042

**Published:** 2024-11-25

**Authors:** Hui Zhong, Jianzhao Zhang, Daming Mo, Hongyu Zheng, Mengting Li, Wenyuan Liu, Xiaoshuang Shen, Xiaomei Cao, Yanbin Jia

**Affiliations:** ^1^ Department of Psychiatry, First Affiliated Hospital, Jinan University, Guangzhou, China; ^2^ Department of Child and Adolescents Psychology, Anhui Mental Health Center, Hefei, China

**Keywords:** major depressive disorder, adolescent, suicide, functional connectivity density, brain imaging

## Abstract

**Background:**

Suicidal behavior including suicidal ideation (SI) and suicide attempts (SA) is a common clinical feature of adolescent patients with major depressive disorders (MDD). We hypothesized that differences in functional connectivity density (FCD) exist between adolescent patients with SA and SI, and aimed to investigate the different suicidal behaviors in adolescents patients with MDD_17_.

**Methods:**

37 MDD adolescents with SA, 34 MDD adolescents with SI, 20 MDD adolescents without SA and SI (non-suicidal group), and 20 adolescents healthy controls (HC) were enrolled in this study. All participants were scanned using functional magnetic resonance imaging (fMRI) to evaluated the FCD. Between-group differences of all variables were analyzed. The relationships between FCD values and clinical scale scores were also analyzed.

**Results:**

The FCD of the left inferior occipital gyrus in the SI group was higher than those in the other groups. The FCD in the SA group was higher than that in the control group. The FCD of the right dorsolateral superior frontal gyrus in the SI group was lower than that in the other three groups. The FCD values of the left precentral gyrus in the SI group were higher than those in the other three groups. The left inferior occipital gyrus FCD positively correlated with the suicide factor score of Hamilton Depression Scale (HAMD), and the right dorsolateral superior frontal gyrus negatively correlated with the HAMD suicide factor score but not with the HAMD total score.

**Conclusion:**

Changes in FCD in adolescent patients with depression and SI can reflect changes in functional connections in the brain.

## 1 Introduction

Major depressive disorder (MDD) is a common psychiatric disorder associated with significant personal suffering and physical and mental disabilities (1). An epidemiological survey in China in 2019 showed that the prevalence of MDD was 6.9% (2). An epidemiological survey of Chinese school students (6-16 years old) in 2022 showed that the prevalence of MDD was 3.2% (3). MDD not only leads to academic difficulties but also increases suicidal risk, leading to long-term social adjustment problems that extend into adulthood and is now one of the leading disease burdens worldwide (4). Particularly, individuals with adolescent-onset depression have a higher suicide risk than those with adult-onset depression (5). According to a study on factors influencing suicide risk in Chinese adolescents published in 2018, adolescents with high scores for depressive symptoms had the highest incidence of suicidal behavior (6); that is, adolescents with MDD had higher suicidal behavior and risk.

Suicidal thoughts and behaviors in youth are major public health concerns. Four forms of suicidal behavior, namely suicidal ideation (SI), suicide plan (SP), suicide attempt (SA), and completed suicide, have been studied. Once SI begins, approximately 60% of attempts occur in 12 months (7). SI, SP, and SA rates among adolescents are 12.1%, 4.0%, and 4.1%, respectively (8). A meta-analysis estimated that the pooled lifetime prevalence rates of SI, SP, and SA were 53.1%, 17.5%, and 23.7%, respectively. Subgroup analyses revealed significant differences in the prevalence of SI and SA between sex and outpatients and inpatients with MDD (9). Moreover, as the frequency and severity of SI increase, the risk of SA and suicide increases (10). In conclusion, a high incidence of MDD associated with suicidal behaviors exists among adolescents.

Specific depressive symptoms such as sad mood, insomnia, concentration problems, and SI are distinct phenomena that differ from each other in important dimensions such as underlying biology, impact on impairment, and risk factors (11). Studies have been conducted on the effects of suicidal behaviors on the brain structure and brain function in MDD with suicidal behavior. A baseline reduction was found in prefrontal volume in patients with mood disorders with subsequent SA (12). Another study found reduced hippocampal and superior temporal gyrus (STG) volumes in young patients with MDD who attempted suicide (13). Adolescents with MDD showed decreased resting-state functional connections between the amygdala seed point the hippocampus and the parahippocampal lobe and enhanced amygdala-precuneus connections; however, this was not associated with suicide severity (14). However, intragroup analysis showed that the severity of suicide positively associated with resting functional connections between the left anterior cuneiform seed point and the left primary motor/somatosensory cortex/superior frontal gyrus and negatively associated with resting functional connections between the left posterior cingulate gyrus seed point, left cerebellum, lateral occipital cortex (OCC), and temporo-occipital fusiform gyrus in adolescents with MDD (15). The amygdaloid-prefrontal and precuneus connections were stronger in the high and attempted suicide groups than in the low SI group, which was stronger than that in the healthy control (HC) group. Stronger connections in the left amygdaloid-rostral anterior cingulate cortex are associated with SA, whereas stronger connections in the right amygdaloid-rostral anterior cingulate cortex are associated with SI (16).

Resting-state functional connectivity (FC) findings highlight reduced positive connectivity between the default mode network (DMN) and salience network in suicide attempters (17). SAs are associated with double cortico-subcortical dissociation in low-frequency amplitude fluctuation (ALFF) values. Decreased ALFF and degree centrality (DC) values, mainly in the frontoparietal network, and increased ALFF values in some subcortical regions distinguished SA from SI. SA may be a distinct subgroup of patients with widespread brain alterations in functional activity and connectivity that could represent vulnerability factors (18). No clear neural differences were identified regarding SI. In a meta-analysis of suicide in MDD, structural magnetic resonance imaging (sMRI) studies suggested that suicide is associated with a reduced volume in the frontal and anterior cingulate gyri, hippocampus, and temporal lobes. The difference in the connections between the default network, significant network, and seed point with the amygdala may be a suicide neuroimaging marker. As we all known, SI is a suicidal thought and state that is associated with depression severity, while SAs are behavior and outcome. The impact they have on current and future suicidal behavior is still poorly studied or not well distinguished. Moreover, different suicidal behaviors in MDD may result in different brain FC. Although, many imaging studies have been conducted on the suicidal behaviors of MDD, and they have verified that suicidal behaviors in patients with MDD are associated with abnormal FC in multiple brain regions. However, no distinction was made between different suicidal behaviors (SI, SA, and no suicide) in adolescents with MDD. Meanwhile, recent studies (19) have also explored the brain network localization of suicide from a brain network perspective, and their rejection of profiles generated by mapping the brain’s intrinsic connectivity network would provide a potential mechanistic framework for understanding human behavior, including suicide.

Functional connectivity density (FCD) was recently developed to identify hub distribution in the human brain. FCD is a graph-theory-based, data-driven approach that does not require any prior assumptions or selection of seed points by measuring the number of functional connections between a single voxel and other voxels in the brain (20). Considering the functional network connection characteristics of a voxel, the higher the FCD value of a particular voxel, the more important the voxel is in the coordinated functional information processing (21). Therefore, FCD provides more information about changes in brain function than functional connectivity (FC).

Few studies have used FCD for depression disorders to investigate MDD in adults using independent component analysis of networks, showing reduced FCD in the mid-cingulate cortex and increased FCD in the OCC. Abnormal FCD in MDD is present in the cingulate cortex and OCC, regarding global FCD (gFCD) (22). Another item for adults with MDD showed significantly decreased short-range FCD in the left STG, right orbital frontal cortex (OFC), and bilateral precuneus, while significantly decreased long-range FCD was found in bilateral middle occipital gyrus (MOG), superior occipital gyrus, and right calcarine (23). A study of patients with depressive disorder with hallucinations found decreased gFCD in the bilateral postcentral gyrus, precentral gyrus, insular cortices, occipital lobe, and increased gFCD in the left middle cingulate cortex (24), and the gFCD in the left inferior temporal gyrus and posterior parahippocampal/hippocampal gyri (PHG/HIP) were associated with insomnia, while the gFCD in the left anterior PHG/HIP correlated with non-insomnia depressive symptoms in the MDD group (25). Depressive disorders and various clinical symptoms (anxiety and insomnia) have corresponding FCD characteristics. The above results indicate that the suicidal behaviors of adolescents with disorders may also have similar manifestations; that is, different clinical characteristics have corresponding FCD manifestations.

Adolescents with MDD associated with suicidal behavior are the focus of clinical attention, and identifying and intervening in SA and SI are important, especially in clinical suicide risk assessment. We hypothesized that differences in FCD exist between MDD adolescents with SA and SI. Therefore, this study aimed to investigate the abnormal FCD between different suicidal behaviors in adolescents with MDD.

## 2 Methods

### 2.1 Participants

This study enrolled patients hospitalized at the Fourth People’s Hospital of Hefei City between December 2022 and December 2023. The inclusion criteria were as follows: (1) meeting the diagnostic criteria for MDD according to the Diagnostic and Statistical Manual of Mental Disorders, IV edition (DSM-IV), (2) being of Han nationality, (3) aged between 13 and 18 years, and (4) no antidepressant medication or psychotherapy was used in the two weeks prior to enrollment, or antidepressant medication was used for less than one week. The exclusion criteria were as follows: (1) concomitant severe physical illness, (2) previous or current history of other DSM-IV Axis I and II episodes, (3) previous history of severe alcohol or drug abuse, and (4) contraindications to magnetic resonance imaging (MRI).

According to the Mini-International Neuropsychiatric Interview (M.I.N.I.), adolescents with MDD were divided into SA (previous SA history), SI (previous SI and non-SA), and non-suicidal (without SA and SI) groups. The SA group included 37 MDD adolescents (7 males and 30 females, the SI group included 34 MDD adolescents (8 males and 26 females), and the non-suicidal group (non-S) included MDD adolescents (4 males and 16 females).

A total of 20 HC (4 males and 16 females) were recruited from Hefei and surrounding communities through local advertisements. The inclusion criteria were as follows: (1) being of Han nationality, aged between 13 and 18 years, and (2) HAMD17 score < 7, no history of mental illness or suicide. The exclusion criteria were as follows: (1) concomitant severe physical illness, (2) previous history of severe alcohol or drug abuse, and (3) contraindications to magnetic resonance imaging (MRI). They were carefully matched with the patient group in terms of age, gender, and years of education.

The Ethics Committee of the Anhui Mental Health Center, Hefei Fourth People’s Hospital, approved this study (no. HSY-IRB-PJ-XJJ-ZH003). After receiving a complete study description, all the participants’ legal guardians provided written informed consent.

### 2.2 Clinical characteristic assessment scales

HAMD-_17_ (21) is a widely used clinical scale for assessing depression symptoms. This scale comprises 17 entries, including 5-factor scores for anxiety/somatization, cognitive impairment, weight, sleep disturbance, blockage, and a final summary factor score. Higher scores indicated more severe depression symptoms experienced by participants during the most recent week.

The Mini-International Neuropsychiatric Interview (M.I.N.I.) (26) is a psychiatrist-administered structured interview that assesses DSM 5th edition. The M.I.N.I. B module assesses suicidal and suicide risks and consists of 15 questions asked by an interviewer and answered yes or no. The first three questions assess recent SAs in the previous month, and the next 11 questions are about SI, plans, or attempts in the past month. The final question asked whether the patient had made any SA.

The Hamilton Anxiety Scale (HAMA): developed by Hamilton to rate the severity of anxiety disorders. It consists of 14 items rated on a scale of 0-4, including two factor scores for somatic and psychogenic anxiety. A total score of more than 29 is probably severe anxiety; more than 21 is definitely significant anxiety; more than 14 is definitely anxiety; more than 7 is probably anxiety; and if less than 7, it indicates no anxiety. The general cut-off score is 14, with higher total scores indicating more severe anxiety in the previous week.

### 2.3 MRI data acquisition

MRI was performed using a 3.0-Tesla MR system (Discovery MR 750w; General Electric, Milwaukee, WI, USA) equipped with a 16-channel head coil. Before scanning, all participants received information regarding the testing procedure and contraindications from the MRI room staff. The participants were instructed to close their eyes, remain quiet and relaxed, stay awake without active thinking, use a tight but comfortable sponge pad to hold their heads in place and lie flat on the scanner bed. Nano-noise-canceling earplugs were used to reduce noise. The scanning sequence and parameters for obtaining high-resolution 3D T1-weighted structural images were as follows: time of echo (TE) = 3.2 ms; time of repeat (TR) = 8.5 ms; flip angle (FA) = 12°; field of view (FOV) = 256 mm × 256 mm; matrix size= 256 × 256; slice thickness (ST) = 1 mm, no gap; voxel size = 1 mm × 1 mm × 1 mm; 188 sagittal slices; and a scanning time of approximately 6 min. Resting-state (Rs) blood oxygenation level-dependent functional MRI (fMRI) data were acquired using a gradient-echo single-echo planar imaging sequence with the following parameters: TE= 30 ms, TR = 2,000 ms, FA= 90°, FOV = 220 mm × 220 mm, matrix size = 64 × 64, ST = 3 mm, slice gap = 1 mm, 35 staggered axial slices, voxel size = 3 mm × 3 mm × 3 mm, 185 volumes, and a scan time of approximately 10 min.

#### 2.3.1 Rs-fMRI preprocessing

Functional data were preprocessed using the Data Processing Assistant of the Rs-fMRI Toolkit (DPARSF; http://rfmri.org/dpabi) (27) and a software package based on Statistical Parametric Mapping software (SPM12; https://www.fil.ion.ucl.ac.uk/spm/). The first 10 volumes were discarded to exclude the influence of unstable longitudinal magnetization. The remaining volumes underwent the following processing steps: slice timing correction, motion correction, co-registering to respective structural images, spatial normalization based on the unified segmentation of structural images, spatial smoothing (Gaussian kernel, FWHM=4 mm), and motion correction via scrubbing. All participants exhibited a maximum displacement of <3 mm and an angular motion of <3°, making them eligible for subsequent analyses.

#### 2.3.2 Calculation of FCD

FCD is based on graph theory. The number of voxels in the brain FCD is the calculation of the functional connections of a given voxel to other voxels in the whole brain. The total number of connections, based on the MATLAB platform, used was, according to Tomasi and Volkow, describing the internal foot of the method written on the Linux platform Ben (28). The FCD for each voxel was calculated and repeated for all the voxels in the brain. Pearson’s linear correlation was performed between two voxels, with a coefficient R>0.6 considered significant. By dividing the brain, the mean voxel was used to increase the normality of the data distribution, giving the FCD a large-scale mean. Subsequently, the time variation for each subject was normalized to the Z-score matrix and finally to normalized image line smoothing (full width 6 mm×6 mm×6 mm under half maximum Gaussian core) (29).

### 2.4 Statistical analysis

Demographic data and clinical characteristics: Descriptive statistical analyses of demographic and behavioral data were conducted using IBM SPSS Statistics software (version 23.0; IBM Corp., Armonk, NY, USA). The one-sample Kolmogorov–Smirnov test was used to assess the normality of continuous variable data, and the measurement data conforming to a normal distribution were expressed as (x ± s). One-way analysis of variance (ANOVA) and chi-square test were used to compare demographic characteristics and clinical psychological scale scores among the groups. Pearson’s correlation analysis was used for correlation analysis between the clinical scales, with statistical significance set at *p* < 0.05.

FCD data analysis: For the images obtained from the Data Processing and Analysis for Brain Imaging statistical analysis, SPM12 software was used to conduct an intergroup one-way ANOVA. Age, sex, and education level were considered as covariates, and one-way ANOVA was employed to compare the four groups of participants, with statistical significance set at *p*<0.05 (adjusted for multiple comparison correction). The different brain regions in the four groups were considered regions of interest (ROI), and the FCD was extracted and averaged. Pearson’s correlation analysis was performed for the FCD of all participants using a clinical assessment scale. Statistical significance was set at *p* < 0.05.

## 3 Results

### 3.1 General information

Participants were matched for sex, age, years of education, and HAMA across the groups, with no statistical differences between the groups (*p* > 0.05). HAMD and suicide factor scores in the SA and SI groups were higher than those in the non-suicide group, and no statistical difference was found between the SA and SI groups (*p* < 0.05). Additionally, no significant difference was observed in the duration of illness between the three depressive subgroups (*p* > 0.05), as shown in **Table 1**.

**Table 1:** Comparison of clinical data and clinical symptom scale scores of the four groups.

Group	HC	SA	SI	Non-suicide	X^2^/F	P
Sex (Male/female)	4/16	7/30	8/26	4/16	0.249	0.969
Education (years)	8.47 ± 1.78	8.21 ± 1.35	7.97 ± 1.46	8.35 ± 1.34	0.573	0.634
Age (years)	15.91 ± 1.92	15.21 ± 1.43	14.97 ± 1.54	15.10 ± 1.29	1.671	0.178
Disease duration (month)	/	18.18 ± 17.01	17.73 ± 13.79	10.73 ± 8.79	1.988	0.143
HAMD_17_	/	21.54 ± 7.47	21.68 ± 6.98	17.08 ± 6.92	3.156	0.047
HAMA	/	20.78 ± 9.86	19.10 ± 8.20	18.88 ± 8.79	0.421	0.658
Suicide factor	/	2.62 ± 1.41	2.75 ± 0.80	0.87 ± 0.67	3.720	0.028

### 3.2 Comparison of FCD values among the groups

**Figure 1:**
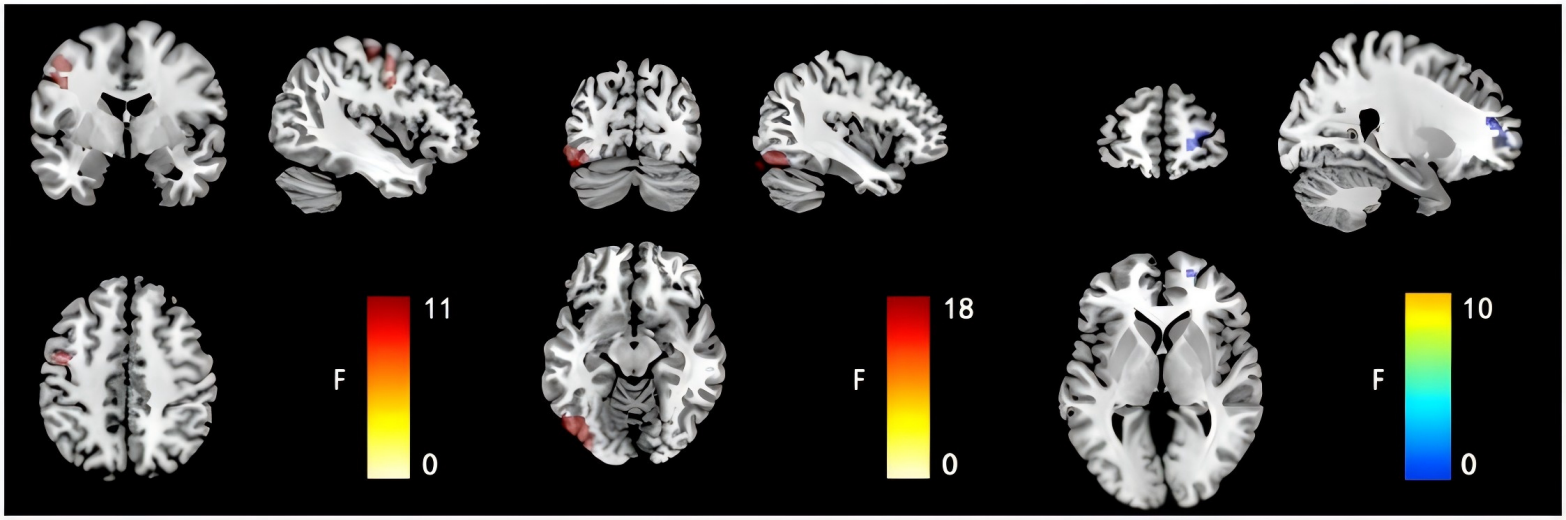
Comparison results of FCD images in the left inferior occipital gyrus, the right dorsolateral superior frontal gyrus and the left cerebral precentral gyrus of the four groups of subjects.

After accounting for the effects of age, sex, and educational level, the FCD values of the groups were compared. The left inferior occipital gyrus FCD was higher in the SI group than in the other groups (SA, non-S and HC group). And the left inferior occipital gyrus FCD was higher in the SA group than in the non-SA group (non-S and HC group) (0.48 ± 0.37 vs. 0.29 ± 0.33 vs. 0.06 ± 0.27 vs. 0.14 ± 0.22, F = 17.969, *p* < 0.05). The right dorsolateral superior frontal gyrus FCD in the SI group was lower than that in the other groups (0.02 ± 0.24 vs. 0.12 ± 0.32 vs 0.21 ± 0.31 vs 0.26 ± 0.29, F = 9.515, *p* < 0.05). The FCD values of the left cerebral precentral gyrus in the SI group were higher than those in the other groups (0.34 ± 0.24 vs 0.17 ± 0.32 vs 0.06 ± 0.24 vs 0.15 ± 0.23, F=10.752, *p* < 0.05), as shown in **Table 2**; **Figure 1**.

**Table 2:** FCD comparison among the four groups.

Brain regions	HC	SA	SI	Non-suicide	F	P
Occipital Inf L	0.14 + 0.22	0.29 + 0.33	0.48 + 0.37	0.06 + 0.27	4.871	0.003
Frontal Sup R	0.26 + 0.29	0.12 + 0.32	0.02 + 0.24	0.21 + 0.31	3.667	0.014
Precentral L	0.15 + 0.23	0.17 + 0.32	0.34 + 0.24	0.06 + 0.24	5.297	0.002

Occipital Inf L, The left inferior occipital gyrus; Frontal Sup R, The right dorsolateral superior frontal gyrus; Precentral L, The Left cerebral precentral gyrus.

### 3.3 FCD correlated with HAMD in the group with adolescent patients with MDD

Further analysis of the correlation between the brain ROI and clinical scales (HAMD total and suicide factor scores) was performed in the depression group. The left inferior occipital gyrus FCD positively correlated with the HAMD scale suicide factor score (*r* = 0.297,*p* = 0.001), and the right dorsolateral superior frontal gyrus FCD negatively correlated with the HAMD scale suicide factor score (*r* = -0.185,*p* = 0.012),but not with the HAMD scale total score (*p* > 0.05), as shown in **Table 3**.

**Table 3:** FCD correlations with clinical scales.

	Occipital Inf L	Frontal Sup R	Precentral L
HAMD(r)	0.043	-0.139	-0.059
P	0.589	0.059	0.425
Suicide factor (r)	0.297	-0.185	0.131
P	0.001	0.012	0.184

Occipital Inf L, The left inferior occipital gyrus; Frontal Sup R, The right dorsolateral superior frontal gyrus; Precentral L, The Left cerebral precentral gyrus.

## 4 Discussion

We explored the characteristics of FCD at different stages or degrees of suicide by dividing the suicidal behaviors of adolescents with depression into SI, SA, and non-suicide groups. This study’s findings can be summarized as follows: (1) The FCD values of different depression subgroups were different. The FCD of the left inferior occipital gyrus and left precentral gyrus in the SI group were higher than those in the other groups, and the FCD of the right dorsolateral superior frontal gyrus in the SI group was lower than that in the other groups. (2) The left inferior occipital gyrus and right dorsolateral superior frontal gyrus FCD correlated with the HAMD suicide factor score but not with the HAMD total score. These results indicate that the FCD in these brain regions was abnormal in the SI group of adolescents with depressive disorder.

Many brain imaging studies exist on depressive disorders associated with suicide. Based on a meta-analysis of 17 studies comprising 381 suicidal individuals and 642 controls, we found increased activity in the STG bilaterally, left middle temporal gyrus, and bilateral MOG, with decreased activity in the right putamen and left insula, were detected in suicidal individuals compared with nonsuicidal individuals. In the subanalyses of suicide attempters and ideators, SA displayed hyperactivity in the STG bilaterally and left middle temporal gyrus and blunt responses in the left insula relative to the controls. Suicidal ideators demonstrated elevated activity in the right MOG and reduced activity in the right putamen compared with the controls. Moreover, increased activity in the right STG, left middle temporal gyrus, and right MOG is associated with higher suicidal ideation scores, revealing several brain regions associated with suicide (30). Youths with high SI with recent attempts showed significantly lower activity in the precentral and postcentral gyri, STG, medial frontal gyrus, insula, and putamen than youths with low SI. In the second analysis, SAs were compared with the other groups. Adolescent SAs showed significantly higher activity in the anterior cingulate cortex and middle frontal gyrus than all other groups (31). Most studies did not strictly distinguish suicidal behaviors such as SAs, SI, and non-suicide in their depressive disorder study or only included SA or suicide ideation.

In this study, we distinguished between different suicidal behaviors, such as SI and SA. We found that the left inferior occipital gyrus and left precentral gyrus gyrus FCD values were greater in the SI group than in the attempted suicide group, but the right dorsolateral superior frontal gyrus FCD values were lower. The left inferior occipital and right dorsolateral superior frontal gyrus FCD were associated with suicide factors but not with the HAMD total score. The inferior occipital gyrus and left precentral gyrus gyrus FCD are characteristic manifestations of SI in adolescents with depressive disorders. In patients with depressive disorders, SI was negatively associated with the resting-state FC in the visual networks, and a significant association was identified between SI and an FC network that included connections between regions in the superior and orbitofrontal cortex, cerebellum, cingulate gyrus including temporal and occipital regions (32). The SI group had a lower dynamic DC (dDC) value than the non-SI group in the left inferior occipital gyrus and a lower voxel mirrored homotopic connectivity value than the non-SI group in the right and left inferior occipital gyrus (33). Regarding dynamic regional activity, the SI group showed a decreased dynamic fractional amplitude of low-frequency fluctuations in the left lingual gyrus and right MOG compared with non-SI. Regarding the dynamic distant connectivity, the SI group showed decreased dDC in the right middle frontal gyrus compared with the non-SI group. Decreased dDC in the right middle frontal gyrus correlates with increased suicidal severity (34). These studies suggest that the occipital and frontal gyri are associated with SI in patients with depressive disorders.

SI is a state in which attempted suicide is the act and result of suicide. Research has shown that SAs are associated with double cortico-subcortical dissociation in the amplitude of low-frequency fluctuations (ALFF). Decreased ALFF and DC values, mainly in the frontoparietal network, and increased ALFF values in some subcortical regions (the hippocampus and thalamus) distinguished SA from suicidal ideators. Compared with the non-SA group, the SA group exhibited increased fractional ALFF in the insula bilaterally and right precentral gyrus. Moreover, the SA group showed increased FC between the right precentral gyrus, left middle frontal gyrus, and left insula compared with the non-SA and HC groups (35). *Post hoc* analyses revealed that SAs exhibited a smaller hemodynamic response in the left precentral gyrus than non-SA and HCs. This suggests that patients with MDD with an SA history demonstrate patterns of verbal fluency task-induced near-infrared spectroscopy signal changes that differ from those demonstrated by individuals without a suicidal behavior history, even in cases where clinical symptoms are similar (36). Compared with non-SAs, SAs with MDD exhibited a larger surface area in the left postcentral and left lateral occipital areas and a larger cortical volume in the left postcentral area (37). These studies showed that suicidal behaviors such as SI and SAs in depressive disorders are correlated with or overlap with many brain regions, such as the occipital area and frontal gyrus. However, this study showed that the inferior occipital gyrus, dorsolateral superior frontal gyrus, and precentral gyrus FCD correlated with SI in adolescents with depressive disorders. FCD is an indicator of the strength of the connection between brain regions.

The DMN and central executive network (CEN) are associated with depressive disorders and brain regions such as the dorsolateral superior frontal, occipital, and central anterior gyri are the components and participating parts of the CEN, DMN, and other networks. Adolescents with depression and SI had lower coherence in the ventral DMN than those without SI. Lower network coherence in all DMN subnetworks was associated with higher SI (38). Another study on teenage suicide showed that suicidal patients showed increased FC in selected DMN regions, such as increased connectivity in the left cerebellum and decreased connectivity in the right posterior cingulate cortex, whereas non-suicidal patients with depression showed increased connectivity in the left superior frontal gyrus, left lingual gyrus, and right precuneus, and decreased connectivity in the left cerebellum (39). The executive control network (ECN) is the core network of cognitive functions. A 2017 study found that reduced FC in the left orbitofrontal thalamic regions (the fronto-thalamic circuits in the left hemisphere) with SI in MDD was inversely proportional to suicidal severity, independent of depression severity. These results suggest that ECN decision-making and information integration are associated with abnormal brain regions in patients with suicidal depression (40). In a study on adolescents with depressive disorders, lower coherence in the left ECN and anterior DMN was independently associated with greater SI severity. When all three significant networks and covariates were included in a single model, only the left ECN significantly predicted SI (41). The abnormality of FCD values, such as in the inferior occipital, dorsolateral superior frontal, and precentral gyri, suggests that the difference in activity in different brain network functional connections may be one of the brain’s functional characteristics of SI.

In addition, this study found that FCD correlated with suicide factor scores but not with HAMD total scores. In a previous study, before and after electroconvulsive therapy, the fALFF in the left superior frontal gyrus and OFC significantly increased and inversely correlated with a reduction in Beck Scale for SI scores, whereas no correlation was found with changes in HAMD-17 scores (42). SI was negatively associated with resting-state FC in visual networks and with fractional anisotropy in the genu of the corpus callosum and right anterior corona radiata (32) and decreased dDC in the right middle frontal gyrus correlated with an increased suicide severity (34). The electroencephalography literature review suggests that the SI and SA may be driven by separate neural circuits (43). These findings suggest that SI in depressive disorders may involve specific brain regions compared with other suicidal behaviors.

This study had some limitations. First, we studied healthy participants and patients with depression with SI or SAs. However, no further distinction was found between SA and SI; that is, SA included some SI. Second, causality could not be inferred from this cross-sectional design, especially when exploring the interactions between psychology and brain imaging. Longitudinal studies or studies with expanded sample sizes that target interventions to improve depression and suicide symptoms should be conducted. Research methods, such as mediation analysis, may be required to determine the direction of causality. Third, the patients had different disease durations, numbers of episodes, and antidepressant treatments involving different medication doses and types, physical therapy (transcranial magnetic stimulation), and psychotherapy. These differences in disease duration and treatment modality may have affected this study’s results. Future studies involving unmedicated patients with initial episodes are required to validate this study’s preliminary results.

## 5 Conclusions

In conclusion, this study demonstrated that suicide ideators can be differentiated from attempters and non-suicidal patients on deficits in global brain activity and connectivity. We observed differences in the left inferior occipital gyrus FCD, right dorsolateral superior frontal gyrus, and left precentral gyrus in adolescents with depression and suicide.

## Data Availability

The raw data supporting the conclusions of this article will be made available by the authors, without undue reservation.
